# Rare Ocular Association With Hailey-Hailey Disease

**DOI:** 10.31486/toj.24.0116

**Published:** 2025

**Authors:** Mamta Singh, Yashdeep Singh Pathania, Praggya Mishra

**Affiliations:** ^1^ Department of Ophthalmology, All India Institute of Medical Sciences Rajkot, Rajkot, Gujarat, India; ^2^ Department of Dermatology, Venereology and Leprology, All India Institute of Medical Sciences Rajkot, Rajkot, Gujarat, India

**Keywords:** *Conjunctivitis*, *corneal neovascularization*, *pemphigus–benign familial*

## Abstract

**Background:**

Hailey-Hailey disease is an autosomal dominant blistering disorder characterized by junctional abnormalities of epidermal keratinocytes. Vesiculobullous eruptions affect the intertriginous areas of individuals with the condition. Ocular involvement associated with Hailey-Hailey disease is rare.

**Case Report:**

A 12-year-old female with a history of recurrent blisters since the age of 3 years presented with concurrent redness, irritation, and watering of both eyes. Slit lamp examination revealed bilateral conjunctival congestion, peripheral corneal neovascularization, lack of corneal luster, and corneal haze. The ocular signs were more prominent in the left eye. Important differentials considered were pemphigus vulgaris and allergic conjunctivitis. However, lack of clinical signs of allergic conjunctivitis, lack of involvement of the oral mucosa, the classic distribution of blisters and their aggravation by triggering factors, the absence of Nikolsky sign, and biopsy of a skin lesion ruled out these 2 diagnoses. The patient was treated with low-potency steroid eye drops, an ocular lubricant, and eye ointment. At her 2-week follow-up examination, the patient exhibited decreased conjunctival congestion, improved corneal luster, and symptomatic relief.

**Conclusion:**

Ocular involvement in Hailey-Hailey disease can lead to chronic ocular inflammation and sequelae, causing a decrease in vision. Our case is noteworthy because of the early onset of Hailey-Hailey disease and the associated ocular manifestations.

## INTRODUCTION

Hailey-Hailey disease, also known as benign familial pemphigus and first described by the brothers Howard and Hugh Hailey in 1939,^1^ is an autosomal dominant rare genodermatosis caused by a mutation of the ATP2C1 gene that results in a defective intracellular calcium pumping mechanism.^2,3^ The disease is characterized by junctional abnormalities of epidermal keratinocytes and clinically presents as chronic recurrent erythematous plaques, bullae, and erosions, principally on the intertriginous regions.^4,5^ Involvement of the eye in Hailey-Hailey disease is uncommon. Oğuz et al documented conjunctivitis and blepharitis in association with Hailey-Hailey disease in a 1997 case report, but their patient's cornea and visual acuity were unaffected.^6^ We report a case of Hailey-Hailey disease with chronic conjunctival congestion, ocular surface inflammation, and corneal neovascularization in a female child.

## CASE REPORT

A 12-year-old female was referred from the dermatology department to the ophthalmology outpatient clinic with the complaints of redness, irritation, and defective vision in both eyes for the prior 9 years. The patient had no symptoms until the age of 3 years, when she started experiencing eye redness and itching. These symptoms were accompanied by multiple blisters in the axillary and perioral area. A general physician treated her skin condition, especially the worsened symptoms in summers, but the patient was unable to provide specific details of treatment. An ophthalmologist treated her progressively increasing eye redness and itching. During the prior 2 years, the patient irregularly used topical steroid eye drops (loteprednol 0.5%) and antiallergic medication (olopatadine 0.1%). She used these medications when her symptoms flared and discontinued them once her symptoms subsided. At the time of presentation to the ophthalmology clinic, she had not used any topical medication for the prior 2 months.

**Figure 1.**
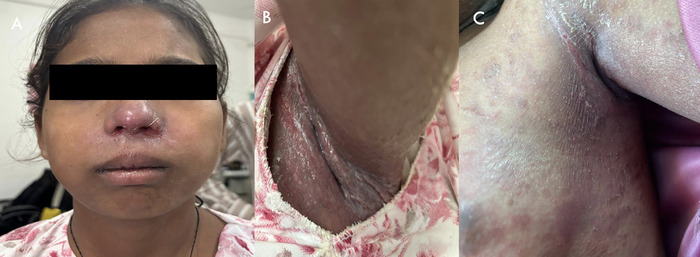
The patient presented with (A) perioral and (B) axillary skin lesions. (C) The axillary lesions on the anterior chest wall improved after 2 weeks of treatment.

When the patient presented to the dermatology department, a clinical diagnosis of Hailey-Hailey disease was established based on the appearance of the lesions, the involvement of the axilla and perioral area (Figures 1A and 1B), and a lesion biopsy. The axillary lesion skin biopsy showed features of epidermal hyperplasia, hyperkeratosis, parakeratosis, acantholysis, and intraepidermal clefts, suggestive of Hailey-Hailey disease. No evidence of involvement of the oral and nasal mucosa was found.

The patient received the standard treatment for Hailey-Hailey disease. She was advised on lifestyle modification and avoiding triggering factors (friction, sweating, hot and humid environments). Topical and systemic antibiotics (mupirocin 2% ointment and oral cefpodoxime 250 mg twice daily) and a topical immunomodulator (tacrolimus 0.1% ointment) were prescribed.

**Figure 2.**
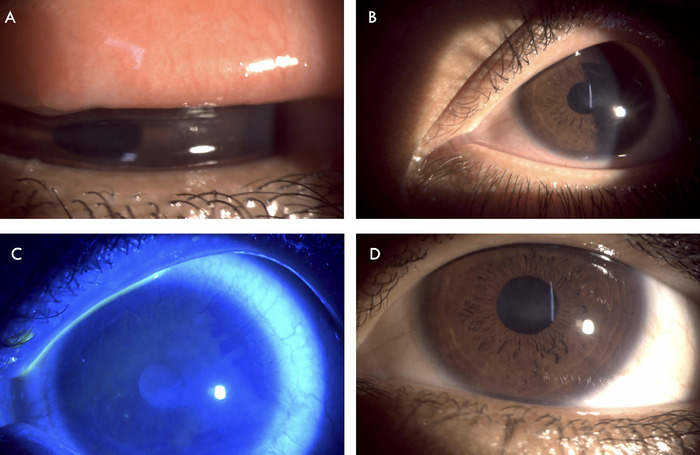
Right eye showed (A) mild edema and congestion of the tarsal conjunctiva; (B) congestion of the bulbar conjunctiva, central corneal haze, and peripheral corneal neovascularization; and (C) punctate staining of the cornea with fluorescein stain. (D) The patient exhibited decreased conjunctival congestion and improved corneal luster after 2 weeks of treatment.

**Figure 3.**
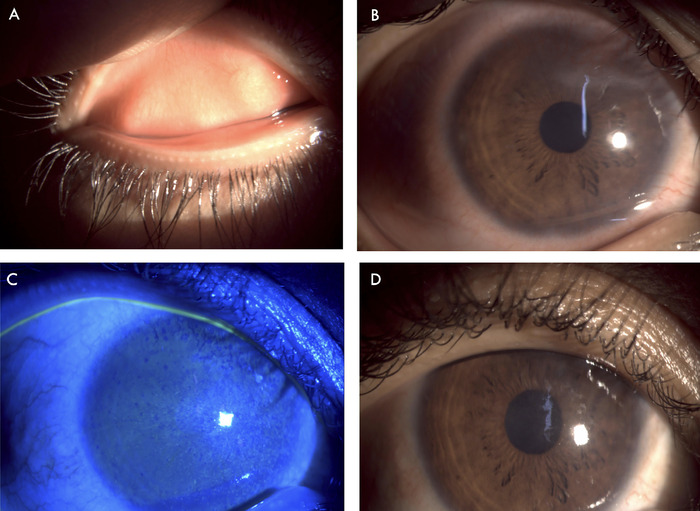
Left eye showed (A) mild edema and congestion of the tarsal conjunctiva, (B) congestion of the bulbar conjunctiva and prominent superior vascularization, and (C) punctate staining of the cornea with fluorescein stain. (D) The patient exhibited decreased conjunctival congestion after 2 weeks of treatment.

At the time of the patient's referral to the ophthalmology clinic, her Snellen visual acuity test result was 6/24 (20/80) in both eyes, improving to 6/6 (20/20) with appropriate spectacle correction. Intraocular pressure measured via noncontact tonometer was 16 mm Hg in the right eye and 14 mm Hg in the left eye. Slit lamp examination of the right eye revealed mild edema and congestion of the tarsal conjunctiva, congested bulbar conjunctiva (grade 1.2 according to the Mandell slit lamp classification system for conjunctival injection^7^), lack of corneal luster, peripheral corneal neovascularization, and central corneal haze (Figures 2A and 2B). Pupillary reaction and posterior segment evaluation were normal. The left eye had the same features as the right eye but with more pronounced conjunctival congestion (grade 2.2^7^) and superior quadrant vascularization of the cornea (Figures 3A and 3B). The tarsal conjunctiva of both eyes showed no evidence of chronic allergic conjunctivitis. Ocular surface staining with 2% fluorescein stain showed punctate corneal stain in both eyes (Figures 2C and 3C). Clinical features were suggestive of chronic ocular surface inflammation, limbal stem cell deficiency, and peripheral corneal neovascularization.

The patient was started on topical low-potency steroid eye drops (loteprednol 0.5%; 1 drop 4 times daily with a weekly taper), an ocular lubricant (combination of polyethylene glycol 0.4% and propylene glycol 0.3%; 6 times daily), and a topical eye ointment (tacrolimus 0.03% at bedtime). Appropriate spectacle correction was recommended.

At the 2-week follow-up evaluation, the patient was symptomatically better. The severity of her skin lesions had decreased (Figure 1C). Ocular examination showed decreased conjunctival congestion (grade 1.17^7^) and improved corneal luster (Figures 2D and 3D). The loteprednol 0.5% eye drops were tapered to twice daily for 1 week, to once daily for another week, and then discontinued. The ocular lubricant and eye ointment were continued at the same frequency for an additional month. The patient was scheduled for a follow-up examination after another 3 weeks of treatment.

## DISCUSSION

The estimated prevalence of Hailey-Hailey disease is approximately 1 in 50,000 individuals.^8^ It commonly manifests in patients between the 2nd and 4th decades, with occasional cases at the extremes of age.^9^ In our case, the patient began developing rashes at age 3 years, with the rashes distributed along the flexural aspects of the body and involving the axillae and anterior aspect of the chest. The perioral area and skin around the nose were involved but the nasal and oral mucosa were not. The genitals were not involved.

As noted earlier, Oğuz et al reported an isolated case of ulcerative blepharitis and conjunctival hyperemia with edema presenting with irritation, burning, tearing, and blurring of vision in a 25-year-old female.^6^ Similarly, our patient presented with chronic intermittent redness, irritation, and watering of both eyes. Considering the chronic intermittent nature of the patient's symptoms, bilateral involvement, and her age, allergic conjunctivitis was an important differential diagnosis. However, slit lamp evaluation did not reveal any clinical evidence of chronic allergic response on the tarsal conjunctiva such as papillae or scarring.

Another important differential diagnosis was pemphigus vulgaris, an autoimmune blistering disease characterized by a preponderance of immunoglobulin G4 anti-desmoglein 3 antibodies.^10^ Desmoglein 3 is also expressed on the ocular surface. Ocular involvement in pemphigus vulgaris is typically unilateral, conjunctivitis and blepharitis are 2 common presentations,^11^ and 80% to 90% of cases have oral and mucosal involvement.^12^ The diagnosis of Hailey-Hailey disease was suggested in our patient because of the absence of Nikolsky sign that ruled out pemphigus vulgaris; the characteristic distribution of blisters and their exacerbation by triggering factors; and the lack of involvement of the oral mucosa. Along with being treated for Hailey-Hailey disease by the dermatologist, the patient was also treated for her ocular surface inflammation, resulting in a decrease in symptoms.

## CONCLUSION

Although rare, Hailey-Hailey disease can be associated with chronic ocular surface inflammation and sequelae. The pathogenesis of ocular involvement in some patients and not in others needs to be evaluated. Our case is notable because of the rarity of Hailey-Hailey disease at such a young age and the associated ocular presentation.

## Notes

*This article meets the Accreditation Council for Graduate Medical Education and the American Board of Medical Specialties Maintenance of Certification competencies for Patient Care and Medical Knowledge.*
